# Towards new human rights in the age of neuroscience and neurotechnology

**DOI:** 10.1186/s40504-017-0050-1

**Published:** 2017-04-26

**Authors:** Marcello Ienca, Roberto Andorno

**Affiliations:** 1 Institute for Biomedical Ethics, University of Basel, Bernouillstrasse 28, 4056 Basel, Switzerland; 2 School of Law, University of Zurich, Zurich, Switzerland

## Abstract

Rapid advancements in human neuroscience and neurotechnology open unprecedented possibilities for accessing, collecting, sharing and manipulating information from the human brain. Such applications raise important challenges to human rights principles that need to be addressed to prevent unintended consequences. This paper assesses the implications of emerging neurotechnology applications in the context of the human rights framework and suggests that existing human rights may not be sufficient to respond to these emerging issues. After analysing the relationship between neuroscience and human rights, we identify four new rights that may become of great relevance in the coming decades: the right to cognitive liberty, the right to mental privacy, the right to mental integrity, and the right to psychological continuity.

*Thou canst not touch the freedom of my mind*

John Milton

## Introduction

The quotation in the epigraph is from the play *Comus*, written by John Milton in 1634. The piece, an exhortation to virtue, follows the story a young noblewoman who has been abducted by a sorcerer called Comus. He has bounded her to an enchanted chair and tried to seduce her with arguments about the charm of bodily pleasure. Despite all his rhetorical assaults, the woman repeatedly refuses his advances and claims that, no matter what he does or says, she will continue to assert her freedom of mind, which is beyond his physical power. In the end, she is rescued by her brothers, who chase off Comus.

The quoted sentence conveys the idea that the mind is a kind of last refuge of personal freedom and self-determination. While the body can easily be subject to domination and control by others, our mind, along with our thoughts, beliefs and convictions, are to a large extent beyond external constraint. Yet, with advances in neural engineering, brain imaging and pervasive neurotechnology, the mind might no longer be such unassailable fortress. As we will explain in this paper, emerging neurotechnologies have the potential to allow access to at least some components of mental information. While these advances can be greatly beneficial for individuals and society, they can also be misused and create unprecedented threats to the freedom of the mind and to the individuals' capacity to freely govern their behavior.

In the research context, brain imaging techniques are widely used to understand the functioning of the human brain and detect the neural correlates of mental states and behavior. Clinical applications of brain imaging as well as other neurotechnologies are significantly improving the well-being of patients suffering from neurological disorders, offering new preventive, diagnostic and therapeutic tools. Outside the clinics, pervasive commercial applications are rapidly providing new possibilities for self-quantification, cognitive enhancement, personalized communication and entertainment for normal users. Furthermore, a number of neurotechnology applications are becoming of major interest in the legal domain, especially tort law, criminal law and law enforcement.

On the other hand, these same technologies, if misused or inadequately implemented, risk creating unparalleled forms of intrusion into people’s private sphere, potentially causing physical or psychological harm, or allowing undue influence on people’s behavior.

This paper makes the case that the possibilities opened up by neurotechnological developments and their application to various aspects of human life will force a reconceptualization of certain human rights, or even the creation of new rights to protect people from potential harm.

In 2013, US President Obama called attention to the potential impact of neuroscience on human rights, emphasizing the need to address questions such as those

“(…) relating to privacy, personal agency, and moral responsibility for one’s actions; questions about stigmatization and discrimination based on neurological measures of intelligence or other traits; and questions about the appropriate use of neuroscience in the criminal-justice system” (Presidential Commission for the Study of Bioethical Issues, 2014).

This article begins by exploring the current possibilities and challenges of neurotechnology, and considers what neurotechnological trends will drive this ethical and legal reconceptualization. After carefully analyzing the relationship between neuroscience and human rights, this paper identifies four new rights that may become of relevance in the coming decades: the right to cognitive liberty, the right to mental privacy, the right to mental integrity, and the right to psychological continuity.

## The neurotechnology revolution

For a long time, the boundaries of the skull have been generally considered the separation line between the observable and unobservable dimension of the living human being. In fact, although primitive forms of neurosurgery used in ancient societies, including pseudo-scientific procedures such as trepanation, could allow for the observation and even manipulation (e.g. selective removal) of brain tissue, yet the neural and mental processes run in the brain and underlying emotions, reasoning and behavior remained at length unobservable. In contrast, modern advancements in neuroscience and neurotechnology have progressively allowed for the unlocking of the human brain and provided insights into brain processes as well as their link to, respectively, mental states and observable behavior. In 1878 Richard Canton discovered the transmission of electrical signals through an animal’s brain. Forty-six years later, the first human electroencephalography (EEG) was recorded. Since then, a neurotechnological revolution has taken place inside and outside the clinics. In the 1990s, sometimes referred to as the ‘decade of the brain’, the use of imaging techniques for neurobehavioral studies increased dramatically (Illes 2003). Today, as a wide and rapidly expanding spectrum of neuroimaging technologies has become clinically and commercially available, the non-invasive recording and display of patterns of brain activity (often associated with the completion of physical or cognitive tasks) has become standard practice. For example, EEG recordings are being widely used to non-invasively measure electrical activity of the brain and detect voltage fluctuations. In addition, derivatives of the EEG technique such as evoked potentials (EPs) and event-related potentials (ERP) allow to average EEG responses to the presentation and processing of stimuli, hence to record brain signals during the performance of specific sensory, cognitive or motor processes. Another technique, functional magnetic resonance imaging (fMRI), allows to measure brain’s electrical activity indirectly, i.e. by using hemodynamic responses (cerebral blood flow) as indirect markers. Current fMRI techniques can localize brain activity, graphically display patterns of brain activation, and determine their intensity by color-coding the strength of activation. fMRI techniques are implemented for a variety of purposes including pre-surgery risk assessment, and functional mapping of brain areas to detect abnormalities (e.g. left-right hemispherical asymmetry in language and memory regions) or to observe post-stroke or post-surgery recovery, as well as the effects of pharmacological and behavioral therapies. In addition, a number of neurological conditions including depression and Alzheimer’s disease can now be diagnosed with the use of fMRI (Koch et al. 2012).

The capacity of neuroimaging techniques to map brain functioning has been tested effective also in gaining insights into people’s intentions, views and attitudes. For example, scientists were able to infer from decoded brain activity which actions participants in their trial were intending to perform. The task in question was to decide whether to add or subtract two numbers and to covertly hold their intention for a few seconds. During that delay, it was possible for scientists to determine with 70% accuracy which of two tasks the subjects were covertly intending to perform (Haynes et al. 2007). In another study, participants toured several virtual-reality houses, and then had their brains scanned while touring another selection. By identifying certain patterns of brain activity for each house, scientists were able to determine which houses their subjects had been to before (Smith 2013). Brain scans do not only allow to ‘read’ concrete experiment-related intentions and memories. They appear even able to decode more general preferences. A US study has shown that fMRI scans can be used to successfully infer the political views of the users by identifying functional differences in the brains of respectively Democrats and Republicans (Schreiber et al. 2013). Similarly, men’s frequent preference for sport cars has been correlated with specific functional differences in the men’s vs the women’s brain (Baron-Cohen 2004).

The possibility of non-invasively identifying such mental correlates of brain functional differences is of particular interest for marketing purposes. Over a decade ago, McClure et al. (2004) used fMRI to show functional differences (increased activation in the dorsolateral prefrontal cortex, hippocampus and midbrain) in the brain of people knowingly drinking Coca Cola as opposed to the same people drinking unlabeled Coke. Their results showed that marketing strategies (e.g. the Coca Cola label) can determine different responses in the brain of consumers (McClure et al. 2004). These results have pioneered the establishment of a spin-out branch of neuroscience at the intersection with marketing research called neuromarketing, which has expanded rapidly over the past decade. Today, several multinational companies including Google, Disney, CBS, and Frito-Lay use neuromarketing research services to measure consumer preferences and impressions on their advertisements or products. In addition, a number of specialized neuromarketing companies including EmSense, Neurosence, MindLab International and Nielsen, routinely apply neuroimaging techniques, mostly fMRI and EEG, but also Steady State Topography (SST) and physiological measurements (e.g. galvanic skin response) to study, analyze and predict consumer behavior. This possibility of *mining the mind* (or at least informationally rich structural aspects of the mind) can be potentially used not only to infer mental preferences, but also to prime, imprint or trigger those preferences. For example, Neurofocus, an American multinational neuromarketing company recently acquired by Nielsen, tested subliminal techniques with the purposes of eliciting responses (e.g. preferring item A instead of B) that people cannot consciously register (Penenberg 2011). These techniques  included embedding stimuli shorter 30 milliseconds, hence under the threshold of conscious perception. In view of these developments, authors have stressed the need to establish ethical and legal standards for neuromarketing practices (Ulman, Cakar, and Yildiz 2015).

Brain imaging techniques were originally developed and are still mostly implemented within the context of clinical medicine and neuroscience research. In recent years, however, a number of neurotechnology applications have made their way onto the market and are now integrated into a number of consumer-grade devices for healthy users with various non-clinical purposes. The umbrella term usually used to encompass all these non-invasive, scalable and potentially ubiquitous of neurotechnologies is “pervasive neurotechnology” (Fernandez, Sriraman, Gurevitz and Ouiller 2015), a notion borrowed from the most widespread notion of pervasive computing. Today, pervasive neurotechnology applications include brain-computer interfaces (BCIs) for device control or real-time neuromonitoring, neurosensor-based vehicle operator systems, cognitive training tools, electrical and magnetic brain stimulation, wearables for mental wellbeing, and virtual reality systems.

Most of these applications use EEG recordings to monitor electrical activity in the brain for a variety of purposes including neuromonitoring (real time evaluation of brain functioning), neurocognitive training (using certain frequency bands to enhance neurocognitive functions), and device control. EEG-based BCIs are being increasingly used as wearable accessories for a number of everyday activities including gaming, entertainment, and smartphone’s remote control. For example, companies Emotiv and Neurosky offer a large assortment of wireless headsets for everyday use that can be connected to compliant smartphones and personal computers (Ienca and Haselager 2016). Brain-control can be used to remotely control several types of devices and engage in several activities including gaming and other forms of entertainment, marketing, self-monitoring and communicating.

The possibility of non-invasive brain control has raised the attention of the mobile communication industry. Several leading companies including Apple and Samsung are incorporating neurogadgets into the accessory assortments of their major products. For instance, iPhone accessories such as the XWave headset already allow to plug directly into compliant iPhones and read brainwaves. Meanwhile, prototypes of next-generation Samsung Galaxy Tabs and other mobile or wearable devices have being tested to be controlled by brain activity via EEG-based BCI (Powell, Munetomo, Schlueter, and Mizukoshi 2013). In the light of these trends, Yuan and colleagues predicted that neurodevices will gradually replace the keyboard, the touch screen, the mouse and the voice command device as humans’ preferred ways to interact with computers (Yuan, Hsieh, and Chang 2010).

Not only neuroimaging devices and BCIs fit into the category of pervasive neurotechnology. Several electrical brain stimulators fit into this category too. Unlike neuroimaging tools, neurostimulators are not primarily used for recording or decoding brain activity but rather for stimulating or modulating brain activity electrically. Portable, easy-to-use, consumer based transcranial direct current stimulation (tDCS) devices are the most widespread form of consumer-grade neurostimulator. They are used in a number of low-cost direct-to-consumer applications aimed at optimizing brain performance on a variety of cognitive tasks, depending on the brain region being stimulated.^1^ Recently, transcranial magnetic stimulation (TMS) - a magnetic method used to briefly stimulate small regions of the brain for both diagnostic and therapeutic purposes, has also evolved into portable devices, which resulted effective in the treatment of migraine (Lefaucheur et al. 2014). Finally, an invasive surgical technique called deep brain stimulation (DBS) involving the implantation of a neurostimulator in the ventrointermediate nucleus of the thalamus has obtained FDA approval and is now increasingly used as a treatment for essential tremor, Parkinson’s disease, dystonia and obsessive–compulsive disorder.

In sum, if in the past decades neurotechnology has unlocked the human brain and made it readable under scientific lenses, the upcoming decades will see neurotechnology becoming pervasive and embedded in numerous aspects of our lives and increasingly effective in modulating the neural correlates of our psychology and behavior. While welcoming continuing progress in neurotechnology development, in this paper we argue that the ethical and legal implications of the neurotechnology revolution should be considered early and in a proactive manner. More in detail, we argue that the legal system has to be adequately prepared to deal with the new challenges that might emerge out of emerging neurotechnology, in particular in the context of human rights. As neurotechnology advances, it is critical to assess whether our current human rights framework is conceptually and normatively well-equipped to face the novel challenges arising at the brain-computer-society entanglement, hence to provide simultaneously guidance to researchers and developers while providing protection to individuals and groups.

## Brain technology and the law

Neuroscience and the law intersect on many levels and on various different issues. This is not surprising. While neuroscience studies the brain processes that underlie human behavior, legal systems are quintessentially concerned with the regulation of human behavior. It is therefore reasonable to claim that both disciplines are destined to be “natural partners” (Goodenough and Tucker 2010). The underlying idea of the new field called ‘neurolaw’ is precisely that better knowledge of the brain will lead to better-designed laws and fairer legal procedures. Examples of potentially legally relevant applications of neurotechnology are numerous. Brain imaging techniques, for instance, might possibly contribute to more evidence-based decisions in criminal justice, from investigation and the assessment of criminal responsibility, to punishment, rehabilitation of offenders, and the evaluation of their risk of recidivism. The tools offered by neuroscience could potentially play also a role in civil law procedures, for example, in the assessment of an individual’s capacity to contract, or of the severity of the plaintiff’s pain in compensation claims. New and more reliable lie detection technologies based on our knowledge of the brain functioning might help to assess the reliability of witnesses. Memory erasure of recidivist violent criminals and of victims of especially traumatic offences (e.g. sexual abuse) is also mentioned as another possibility opened by our new knowledge of the brain (Goodenough and Tucker 2010).

A possibly game-changing use of neurotechnology in the legal field has been illustrated by Aharoni et al. (2013). In this study, researchers followed a group of 96 male prisoners at prison release. Using fMRI, prisoners’ brains were scanned during the performance of computer tasks in which they had to make quick decisions and inhibit impulsive reactions. The researchers followed the ex-convicts for 4 years to see how they behaved. The study results indicate that those individuals showing low activity in a brain region associated with decision-making and action (the Anterior Cingulate Cortex, ACC) are more likely to commit crimes again within 4 years of release (Aharoni et al. 2013). According to the study, the risk of recidivism is *more than double* in individuals showing low activity in that region of the brain than in individuals with high activity in that region. Their results suggest a “potential neurocognitive biomarker for persistent antisocial behavior”. In other words, brain scans can theoretically help determine whether certain convicted persons are at an increased risk of reoffending if released.

This prospect evokes Philip Dick’s 1956 science fiction story “The Minority Report”, which was adapted into a movie in 2002. The plot is about a special police unit (“Precrime Division”) which is able to identify and arrest murderers before they commit their crimes. The system is believed to be flawless until an officer from that same unit is mistakenly accused of a future murder (Dick 2002). This dystopian scenario, which could result from the new knowledge about the brain, raises important ethical and human rights questions. How much evidence is needed to prove that brain scans are likely to flag only the truly high risk offenders? Can neurotechnology-generated data, which have a probabilistic nature, be straightforwardly applied to predict the criminal behavior of a particular individual? Can these preliminary findings, which were based on a very specific cohort, be generalized to other groups? In any case, it is clear that much more work is needed to ensure the reliability of the technique before authorizing its use by courts, certainly not as a substitute for current methods for dangerousness assessment, but maybe as an additional, complementary tool.

Other brain technologies that may be relevant for the legal system are lie detectors, mental decoders, and brain printers. Lie detectors are devices capable to record and measure brain responses associated with the retrieval of memories, with the purpose of ascertaining the truth-values of statements relative to those memories. Traditional lie detectors, like the polygraph, measure some bodily markers such as blood pressure, heart rate, and muscular reactions. Despite their low reliability, they are regularly used by some government agencies to screen their employees. However, they are very rarely accepted as evidence in US courts. The new generations of lie detectors, which are EEG-based and fMRI-based, are regarded as much more reliable than the polygraph, as they detect the lie at its source: the brain. In the United States, at least two companies -*No Lie MRI* and *Cephos Corp* - are currently offering fMRI lie-detection services (Greely 2009). A study published in 2005 by a research group linked to Cephos, claimed that fMRI-based lie detection has a reliability of around 90%. The study predicted that the procedure will be further improved and ready to be used in courts in the not too distant future (Kozel et al. 2005). More recent studies have confirmed the higher reliability of fMRI-based lie detectors compared to polygraphy (Langleben et al. 2016). In parallel, mental decoders are capable of decoding mental states and translating them into observable outputs such as text, verbal signals or graphic images. For example, Herff et al. (2015) and Mirkovic et al. (2015) have independently demonstrated the effectiveness of a decoder capable of reconstructing speech from brain waves (Herff et al. 2015; Mirkovic, Debener, Jaeger, and De Vos 2015). Such devices have a great potential for clinical applicability as they could benefit several classes of neurological patients, especially those suffering from locked-in syndrome and paralysis. Such patients, who might have lost their capacity to produce verbal communication, would be enabled to re-interact with the external world by producing speech solely by brain activity. Outside the clinical setting, such decoders are tested to enhance mobile communication through thought-to-text converters. Not all mental decoders are designed to enhance users’ autonomy. Some devices are currently tested for monitoring brain states with the purpose of guiding the individual’s behavior. For example, NASA and Jaguar are jointly developing a technology called *Mind Sense*, which will measure brainwaves to monitor the driver’s concentration in the car (Biondi and Skrypchuk 2017). If brain activity indicates poor concentration, then the steering wheel or pedals could vibrate to raise the driver’s awareness of the danger. This technology can contribute to reduce the number of accidents caused by drivers who are stressed or distracted. However, it also opens theoretically the possibility for third parties to use brain decoders to eavesdropping on people’s states of mind.

Similar implications are raised by brain printers. These are prototypical devices that are currently tested as brain-based authentication methods. For example, researchers at Binghamton University in the state of New York have devised a way to verify a person’s identity based on how their brain responds to certain words. The researchers observed the brain signals of 45 volunteers as they read a list of 75 acronyms, such as FBI and DVD, and recorded the brain’s reaction to each group of letters, focusing on the part of the brain associated with reading and recognizing words. It turns out that participants’ brains reacted differently to each acronym - so that a computer system was able to identify each volunteer with 94% accuracy (Armstrong et al. 2015). This technology, which could in the short term replace passwords and fingerprints as authentication tool for personal accounts, raises novel privacy and security issues.

As neurotechnology advances and opens novel opportunities for monitoring and controlling cognitive function, there is uncertainty on how the law should cope with such advancements. In particular, it remains debatable whether emerging trends in neurotechnology call for a revision or even a replacement of existing legal concepts at various levels including civil law, tort law, business law and legal philosophy. While increasing attention is being devoted in the literature to emerging neurotechnology applications in the context of criminal law or to the increasing use of neuroscience evidence in courts, little focus has been directed to the implications of advancing neuroscience and neurotechnology for human right law. This neglected component of the neurolaw discourse is of particular relevance since the universal nature of the human right framework could provide a solid foundation for this emerging ‘jurisprudence of the mind’.

## Neuroscience and human rights

### Overview

While neurotechnology has the potential to impact human rights such as privacy, freedom of thought, the right to mental integrity, the freedom from discrimination, the right to a fair trial, or the principle against self-incrimination, yet international human rights law does not make any explicit reference to neuroscience. In contrast to other biomedical developments, which have already been the subject of standard-setting efforts at the domestic and international level, neurotechnology still largely remains a *terra incognita* for human rights law. Nonetheless, the implications raised by neuroscience and neurotechnology for inherent features of human beings, urge a prompt and adaptive response from human rights law.

The adaptive ability that human rights law has shown in responding to the challenges posed by genetic technology may help to anticipate how this branch of law could evolve in the coming years in response to new issues raised by neuroscience. Since the end of the 1990s, the international community has made significant efforts to address a great variety of issues that result from the increasing access to human genetic data. In 1997, the *Universal Declaration on the Human Genome and Human Rights* (UDHGHR) was adopted to prevent that genetic information is collected and used in ways that are incompatible with respect for human rights, and to protect the human genome from improper manipulations that may harm future generations. The principles contained in this instrument were further developed in 2003 by the *International Declaration on Human Genetic Data* (IDHGD), which sets out more specific rules for the collection of human biological samples and genetic data. It is interesting to note that from the interaction between genetics and human rights resulted entirely new rights, such as the ‘right not to know one’s genetic information’, which is formally recognized by the UDHGHR (Art. 5(c)) and the IDHGD (Art. 10), as well as by other international and national regulations. In addition to the recognition of new rights, ‘old’ rights -such as the right to privacy and the right against discrimination- were specifically adapted to the novel challenges posed by genetics. This close connection between life sciences and human rights was further strengthened by the 2005 Universal Declaration on Bioethics and Human Rights, which comprehensively addresses the linkage between both fields (Andorno 2013). This latter document sets out principles that are applicable not only to genetics but to other biomedical and life sciences issues.

In this paper we claim that, similarly to the historical trajectory of the ‘genetic revolution’, the ongoing ‘neuro-revolution’ will reshape some of our ethical and legal notions. In particular, we argue that the growing sensitivity and availability of neurodevices will require in the coming years the emergence of new rights or at least the further development of traditional rights to specifically address the challenges posed by neuroscience and neurotechnology. This argument is in accordance with the observation of how human rights have historically emerged and developed in modern societies. Human rights, in fact, have always arisen as specific responses to recurrent threats to fundamental human interests (Nickel 1987), to human dignity (Habermas 2010), or to what is required by a “minimally good life” (Fagan 2005). As we attempt to show in this paper, the individual quest to exert control over one’s own neuro-cognitive dimension as well as the emergence of potential threats to basic human goods or interests posed by the misuse or inadequate application of neurotechnological devices may require a reconceptualization of some traditional human rights or even the creation of new neuro-specific rights.

It goes beyond the scope of this article to discuss the different theories about the foundations of human rights, or to take a position in this regard. For the purposes of our investigation we chose to adopt a broad practical conception of human rights, like the one proposed by Beitz (2011, p. 109), who argues that they are “requirements whose object is to protect urgent individual interests against predictable dangers (‘standard threats’) to which they are vulnerable under typical circumstances of life in a modern world order composed of states” (Beitz 2011). In general terms, it can be said that the scope of human rights is to guarantee both the necessary negative and positive prerequisites for leading a minimally good life (Fagan 2015).

A common objection against the recognition of new rights is that it leads to the so-called ‘rights inflation’, which is the objectionable tendency to label everything that is morally desirable as a ‘human right’. The unjustified proliferation of new rights is indeed problematic because it spreads skepticism about all human rights, as if they were merely wishful thinking or purely rhetorical claims. Rights inflation is to be avoided because it dilutes the core idea of human rights and distracts from the central goal of human rights instruments, which is to protect a set of truly fundamental human interests, and not everything that would be desirable or advantageous in an ideal world.

A frequently accepted way to avoid rights inflation is to impose justificatory tests for specific human rights. For example, according to Nickel (2014), it could be required that a proposed human right should not only deal with some very important good but also respond to a common and serious threat to that good, impose burdens on the addressees that are justifiable and no larger than necessary, and be feasible in most of the world’s countries (Nickel 2014). The international law scholar Philip Alston (1984) has suggested a list of criteria that a given claim must satisfy in order to qualify as a ‘human right’ in terms of international law. In his view, the proposed new human right must “reflect a fundamentally important social value”; “be consistent, but not merely repetitive, of the existing body of international human rights law”; “be capable of achieving a very high degree of international consensus”, and “be sufficiently precise as to give rise to identifiable rights and obligations”.

For the reasons we give below, we think that the new rights advocated in this paper − the right to cognitive liberty, the right to mental privacy, the right to mental integrity, and the right to psychological continuity − fulfill these requirements and therefore do not raise the risk of rights inflation.

This proposal of neuro-specific human rights is consistent with Glen Boire’s advocacy of a “jurisprudence of the mind” that “takes account of the latest understandings of the brain” and “which situates these within our country’s tradition of embracing individual, self-determination and limited government” (Boire 2003, p. 10). As brain technology is rapidly reshaping the infosphere and the digital infrastructures in our societies, there is an urgent need to proactively assess whether our current ethical and legal frameworks are ready to face this emerging scenario.

At this stage it is also worth noting that many of the issues discussed in this paper are not unique to cutting-edge neurotechnology but have precedents in more traditional interventions. For example, breaches for mental privacy emerged before the invention of neuroimaging and neuromonitoring technologies through more rudimental techniques such as interrogation and polygraph-based lie detection. These interventions, however, do not target neural processing directly but only via proxy-processes such as speech, behavior, and physiological indices (e.g. pulse and skin conductivity). In addition, the degree of accuracy and resolution of such techniques is remarkably low (Iacono 2008), hence often insufficient to support epistemologically justified inferences about mental information. Similarly, threats to mental integrity and psychological continuity were posed by non-computational interventions such as psychoactive drugs and hypnotic inductions way before the invention of neurostimulation and brain-machine interfacing. However, these techniques are often characterized by limited efficacy and reliability in purposively manipulating mental activity as well as low degrees of selectivity in targeting neural processes. Based on these considerations, we argue that advanced neurotechnology enables a degree of access into and manipulation of neural processes significantly higher than other techniques. Therefore, while we consider the ethical and legal analysis presented in this paper applicable to the entire spectrum of both computational and non-computational brain interventions, we argue that the degree of perturbation of advanced neurotechnology on the current ethical-legal framework is quantitatively higher than non-computational techniques. For this reason we situate neurotechnology as the focus of our proposed normative upgrade.

### Cognitive liberty

A first, essential step towards the creation of a neuro-oriented human rights framework has been the recent debate over the notion of cognitive liberty. According to Bublitz (2013), this complex notion, often also referred to as *mental self-determination*, comprises two fundamental and intimately related principles: (a) the right of individuals to use emerging neurotechnologies; (b) the protection of individuals from the coercive and unconsented use of such technologies. As he concisely put it, cognitive liberty is the principle that guarantees “the right to alter one’s mental states with the help of neurotools as well as to refuse to do so” (Bublitz 2013, p. 234).

Proponents of cognitive liberty suggest considering it a “fundamental human right” as well as “a central legal principle guiding the regulation of neurotechnologies” (Ibid.). The reason of its fundamental function stems from the fact that “the right and freedom to control one’s own consciousness and electrochemical thought processes is the necessary substrate for just about every other freedom” (Sententia 2004). In fact, as Bublitz argued, “it is hard to conceive any conception of a legal subject in which the mind and mental capacities (e.g. acting from reasons, deliberation) are not among its necessary constitutive conditions” (2013, p. 242). Cognitive liberty, therefore, is necessary to all other liberties, because it is their neuro-cognitive substrate. As such, cognitive liberty resembles the notion of ‘freedom of thought’ which is usually considered the essential justification of other freedoms such as freedom of choice, freedom of speech, freedom of press, and freedom of religion. Not surprisingly, Sententia (2004) presented cognitive liberty as a *conceptual update* of freedom of thought that “takes into account the power we now have, and increasingly will have to monitor and manipulate cognitive function”. Some legal scholars such as Boire and Sententia have interpreted the right to cognitive liberty with special focus on the protection of individual freedom and self-determination from the State. For example, Sententia has claimed that “the State cannot, consistent with the First Amendment of the Constitution, forcibly manipulate the mental states, and implicitly the brain states of individual citizens”.

Given its conceptual complexity, cognitive liberty is multi-dimensional. Bublitz recognizes at least three “interrelated but not identical dimensions” (Bublitz 2013, p. 251). These are: (i) the liberty to change one’s mind or to choose whether and by which means to change one’s mind; (ii) the protection of interventions into other minds to protect mental integrity, and (iii) the ethical and legal obligation to promoting cognitive liberty. These three dimensions configure cognitive liberty as a complex right which involves the prerequisites of both negative and positive liberties in Berlin’s sense (Berlin 1959): the negative liberty of making choices about one’s own cognitive domain in absence of governmental or non-governmental obstacles, barriers or prohibitions; the negative liberty of exercising one’s own right to mental integrity in absence of constrains or violations from corporations, criminal agents or the government; and finally, the positive liberty of having the possibility of acting in such a way as to take control of one’s mental life.

Being the neurocognitive substrate of all other liberties, cognitive liberty cannot be reduced to existing rights, hence is immune to the risk of rights inflation. In addition, since cognitive life, although in various forms and degrees, is inherent in all human beings, cognitive liberty is consistent with a definition of human rights as inalienable fundamentals rights “to which a person is inherently entitled simply because she or he is a human being” (Sepuldeva, Van Banning, and van Genugten 2004), regardless of their nation, location, language, religion, ethnic origin or any other status. Consequently, its integration into the human right framework would enable the protection of constitutive features of human beings that are not being entirely protected by existing rights.

For the purposes of our analysis, in this article we will focus exclusively on the negative formulation of the right to cognitive liberty, namely as the right to refuse coercive uses of neurotechnology. In addition, while we welcome the introduction of the right to cognitive liberty, we argue that this notion is not sufficient alone to cover the entire spectrum of ethical and legal implications associated with neurotechnology. Rather, the establishment of cognitive liberty as a human right should be coordinated with a simultaneous reconceptualization of existing rights or even the creation of other new neuro-specific rights. These are the right to mental privacy, the right to mental integrity and the right to psychological continuity.

### The right to mental privacy

#### The right to privacy

Today’s infosphere is more intrusive than at any other time in history. Websites regularly use cookies to record store visitors’ information such as browsing activities, preferences, personal data, visited pages, passwords, credit card numbers, etc. Big and small corporations engage in data-mining activities that capture massive amounts of data about users. Much of this information is about daily activities: what was purchased, when, where and how much was paid. E-mail accounts are stuffed with advertisements and unsolicited offers. Phone numbers and personal addresses are captured in databases and sold to corporations and government agencies. In addition, video surveillance, facial recognition technology, spyware are opening up people’s daily activities for public consumption. As Moore (2010) puts it, today “informational privacy is everywhere under siege”.

The widespread availability of neurotechnology applications will provide multiple opportunities for individuals to access and exert control over their brain-activity, hence resulting in a number of potentially beneficial activities such as self-monitoring, neuro-enhancement, and brain-controlled computer use. However, these same tools will disseminate an unprecedented volume and variety of brain information outside the clinical domain and potentially increase the availability of such information to third parties. As pervasive applications of neurotechnology are introducing brain data into the infosphere, they are thereby exposing them to the same degree of intrusiveness and vulnerability to which is exposed any other bit of information circulating in the digital ecosystem. At present, no specific legal or technical safeguard protects brain data from being subject to the same data-mining and privacy intruding measures as other types of information. In the words of Nita Farahany, “there are no legal protections from having your mind involuntarily read”.^2^ The reason for that stems from the fact, as Charo (2005) observes, that “technology innovates faster than the regulatory system can adapt”.

A large number of ethical, legal, and social questions arise from these neurotechnological possibilities. These include: For what purposes and under what conditions can brain information be collected and used? What components of brain information shall be legitimately disclosed and made accessible to others? Who shall be entitled to access those data (employers, insurance companies, the State)? What should be the limits to consent in this area?

Although a first attempt of response to these questions can be made by appealing to existing legal norms, we claim that specific legal notions and provisions have to be developed. The first notion involved in these debates is that of *privacy*. International human rights law formally recognises the right to privacy. The Universal Declaration of Human Rights (UDHR) states that “no one shall be subjected to arbitrary interference with his privacy, family, home or correspondence, nor to attacks upon his honour and reputation. Everyone has the right to the protection of the law against such interference or attacks” (Article 12). Similarly, the 1950 European Convention on Human Rights (ECHR) stipulates that “everyone has the right to respect for his private and family life, his home and correspondence” (Article 8 para 1). It is interesting to note that the right to privacy is one of the few rights that was recognized by international law as a broad, umbrella right before it was included in any state constitution (Diggelmann and Cleis 2014).

At the European level, the right to privacy recognized by the ECHR was developed by the 1995 EU Data Protection Directive (95/46/EC), which specifically aims at protecting individuals with regard to the processing and transfer of personal data. Currently, the EU is planning to adapt the data protection rules to the challenges to privacy posed by the new digital environment. The overall goal of the upcoming Directive and Regulation is to empower individuals with more control over their personal data.^3^ Also the EU Charter of Fundamental Rights, adopted in 2000, states the general right to protection of private life in Article 7 and specifies in Article 8 that “everyone has the right to the protection of personal data concerning him or her” (para 1). According to paragraph 2 of the latter provision,

“[s]uch data must be processed fairly for specified purposes and on the basis of the consent of the person concerned or some other legitimate basis laid down by law. Everyone has the right of access to data which has been collected concerning him or her, and the right to have it rectified”.

The first question that arises in the context of the current privacy protection standards is whether the traditional right to privacy also covers the data contained in and generated by our minds. An answer to this dilemma is not immediately available, not least because there is no consensus in the legal literature on a definition of privacy. This can be explained by the disparate content of this right, which includes not only the right to control access to personal information, but also to our bodies and to specific private places. In their seminal article, published in 1890, Samuel Warren and Louis Brandeis articulated the right to privacy as “a right to be let alone” (Warren and Brandeis 1890). Their primary concern was the increasing interest of the yellow press in gossiping and revealing personal information about individuals, including pictures of private persons without their consent. This specific instance of privacy was further developed by Alan Westin and other authors into the broader notion of informational privacy, i.e. the control over information about oneself. According to Westin, privacy can be described in terms of our claim to determine for ourselves when, how, and to what extent information about us is communicated to others (Westin 1968). Today, the “right to be let alone” delineated by Warren and Brandeis more than one century ago has clearly become relevant to areas far removed from their original concerns. The various facets of the modern understanding of privacy continue to expand as technological developments continue. Neuroscience is very likely to become in the near future one of the new areas in which the right to privacy is called to play a fundamental and unexpected role.

#### The emergence of a right to mental privacy

Science fiction can be very helpful to anticipate the challenges that science and technology may pose in the future, as well as the possible responses to them. In a *Star Trek* novel written in 1990, Captain Kirk has been informed that a dangerous spy has surreptitiously joined one of the groups that are visiting the spaceship *Enterprise*. Kirk desperately wants to identify the intruder and to know more about him and his plans. By appealing to one of his staff members who has telepath abilities, Kirk wants to read the minds of all the visitors. However, the Captain is reminded by one of his assistants that, according to the law, “the right to mental privacy is an inalienable right of all Federation citizens and shall not be abrogated without due process of law” (Mitchell 1990). Moreover, “to find one guilty individual in either of those groups means there is a large probability of invading the privacy of a number of innocent people” (Ibid., p. 150).

The kind of dilemmas described in this futuristic scenario, which is set in the 23rd-century, may become a reality much earlier than expected. Developments in neuroimaging, like those mentioned above, have raised concerns about the ethics and legality of ‘mind-reading’. It is true that functional brain imaging cannot really ‘read’ thoughts, but can only highlight differences between brain activations during different cognitive tasks, and to infer from such differences certain conclusions about an individual’s thoughts. However, the fact remains that, even if in an indirect manner, these new tools are increasingly able to determine with a high degree of accuracy certain brain data that belong to the private sphere and deserve to be protected from public scrutiny.

In modern societies, privacy and data protection norms cover the use and disclosure of various kinds of personal information. Since the data decoded from an individual’s brain can be regarded as ‘personal information’ − or ‘personally identifiable information’, as it is called in the US−, there is in principle no reason why such data could not be covered by existing privacy and data protection regulations. If one has a “reasonable expectation of privacy”^4^ regarding the identifying information derived from one’s blood or saliva samples, surely one has a reasonable expectation of privacy regarding the data decoded from one’s own mind (Shen 2013).

However, the special nature of brain data, which relate very directly to one’s inner life and personhood, and the distinct way in which such data are obtained, suggest that specific safeguards will be probably needed in this domain. It should be noted that traditional privacy rules seek to safeguard ‘external’ information about people.

The particularity of brain data is that the *information* to be protected is not easily distinguishable from the *source* itself that produced the data: the individual’s neural processing. This is what we can call the “inception problem”, which complicates the analysis of the issues at stake when traditional approaches to privacy are used. In other terms, the neurotechnological future we are approaching will require us to guarantee protection not only to the information we record and share, but also to the *source* of that information since they may be inseparable. In order to implement this we would need wider privacy and data protection rights that can be also applied at a higher and chronologically antecedent level: *our neural activity*.

An additional reason for concern about privacy in this domain is that brain signals allow to distinguish or trace an individual‘s identity and are potentially linkable to that individual. Some brain records (e.g. EEG-recorded signals) can be used as a unique biometric identifier, similarly to fingerprints or DNA. Back in 2007, Palanippan and colleagues developed a EEG based biometric framework for automatic identity verification (Palaniappan and Mandic 2007). Since then, a huge number of unobtrusive EEG-based biometric systems have been developed for the purposes of individual recognition (Campisi, La Rocca, and Scarano 2012; La Rocca, Campisi, and Scarano 2012), person authentication (Marcel and Del Millan 2007; Palaniappan 2008), and person identification (Brigham and Kumar 2010; Mohammadi, Shoushtari, Molaee Ardekani, and Shamsollahi 2006). However, unlike other identifiable information, brainwaves can be potentially recorded without individual’s awareness, and therefore in absence of a real ability of the person to consent to the collection and use of that information. With the growing market of portable EEG-based neuroheadsets and in absence of a real possibility for obtaining informed consent for the processing of the records they generate, there is a need for the law to lay down new protective responses to the processing of brain data. The need to protect information generated below the threshold of voluntary control demands for the recognition of a new right that is specifically tailored on the characteristics of brain information and the new possibilities opened by mind-reading technologies.

In the light of the emerging neurotechnologies, it is also necessary to explore the -technical and legal - possibility of applying a filter to the flow of brain information with the purpose of distinguishing the information we consciously want to keep private from the one we want to disclose publicly. In the current information society we are constantly required to draw a distinction between private and public information: for example, when we set up the contact page on our website or when we decide with whom to share our mobile phone number. The basic psychological assumption that underlies this phenomenon is that competent adults have the psychological capacity to consciously filter the information flow and reasonably identify the bits of information that must be kept private. Privacy, in fact, is both a right and an ability. As an ability, it enables individuals or groups to seclude themselves, or information about themselves, and thereby express themselves selectively. This idea has been widely imported into the information technology sphere, where privacy is often described as the ability (or perceived ability) to control submitted personal information -especially when using the Internet (Dinev and Hart 2004). In order to exercise this ability meaningfully we need a rational medium that is capable to filter the information flow and decide what to disclose. This medium is thought, as well captured by the famous adagio in computer security “the best anti-virus software is the brain”.

Based on these specific challenges, we argue that current privacy and data protection rights are insufficient to cope with the emerging neurotechnological scenarios. Consequently, we suggest the formal recognition of a right to mental privacy, which aims to protect any bit or set of brain information about an individual recorded by a neurodevice and shared across the digital ecosystem. This right would protect brainwaves not only as data but also as data generators or sources of information. In addition, it would cover not only conscious brain data but also data that are not (or are only partly) under voluntary and conscious control. Finally, it guarantees the protection of brain information in absence of an external tool for identifying and filtering that information. In short, the right to brain privacy aims to protect people against illegitimate access to their brain information and to prevent the indiscriminate leakage of brain data across the infosphere.

It is worthy of mention that violations of mental privacy can occur also in absence of direct intrusion into the victim’s neural processing. For example, brain data collected for research purposes are usually stored for analysis on externally located EEG-databases and repositories. Similarly brain-data generated by consumer-grade brain-computer interfaces (BCI) are sent to a connected app and can be stored in the cloud or other data store end points. In either case, these data can be accessed also in absence of the person who generated those data and without intervening into the person’s brain signaling.

#### Is the right to mental privacy an absolute or a relative right?

Most human rights, including privacy rights, are *relative*, in the sense that they can be limited in certain circumstances, provided that some restrictions are necessary and are a proportionate way of achieving a legitimate purpose.^5^ In specifically dealing with the right to privacy, the European Convention on Human Rights states that this right admits some restrictions “for the prevention of disorder or crime, for the protection of health or morals, or for the protection of the rights and freedoms of others” (Art. 8, para 2). Only very few rights, such as the freedom of thought, freedom from slavery, torture and inhuman or degrading treatment or punishment are regarded by international human rights law as not subject to any exceptions and, therefore, as *absolute rights*. In which of both categories should the right to mental privacy be placed? Can nonconsensual intrusions into people’s brain data be justified in certain circumstances or should be unconditionally banned? More concretely, does the right to mental privacy protect individuals from being compelled by courts or the state to brain-based interrogations?

Paul Root Wolpe has suggested that due to fears of government oppression, we should draw a bright line around the use of mind-reading technologies:

“The skull should be designated as a domain of absolute privacy. No one should be able to probe an individual’s mind against their will. We should not permit it with a court order. We should not permit it for military or national security. We should forgo the use of the technology under coercive circumstances even though using it may serve the public good” (Wolpe 2009).

Similarly, it has been argued that “nonconsensual mind reading is not something we should never engage in” (Stanley 2012). The claim is that mind-reading techniques constitute “a fundamental affront to human dignity” (Ibid). Consequently, “we must not let our civilization’s privacy principles degrade so far that attempting to peer inside a person’s own head against their will ever become regarded as acceptable” (Ibid).

Are these calls for an unconditional ban on compulsory mind-reading justified? Or could this procedure be acceptable in certain circumstances (for instance, when faced with a serious crime or a terrorist attack)? As mentioned above, privacy rights are not absolute, but relative. The collection, use and disclosure of private information is permissible when the public interest is at stake. For example, in many jurisdictions, compulsory genetic testing can be undertaken to attempt to identify criminal offenders. Considering the non-invasive and painless nature of brain-scans, there are prima facie good reasons for thinking that their nonconsensual use would be justified, with a court warrant, under special circumstances when there are reasonable grounds to believe that an individual has committed a serious crime or is involved in the planning of a serious crime.

However, this dilemma becomes more intricate when it is seen not in connection to privacy issues, but in the light of the principle of prohibiting coerced self-incrimination. This problem particularly arises when the results of brain scans are regarded not as mere *information* about individuals (such as buccal or blood-derived DNA, fingerprints, etc.), but as a *testimony* because in this latter case the self-incrimination clause would enter into play.

The ban on coerced self-incrimination is widely recognized across the democratic world as being an integral component of a fair criminal justice. This privilege is a logic consequence of the presumption of innocence, which places the burden of proof of guilt on the prosecution. In other words, people suspected of a crime do not have any obligation to assist in providing evidence against themselves. The privilege against self-incrimination is very closely related to the right to remain silent and can overlap with it. However, there is a conceptual difference between them: while the former concerns the threat of coercion in order to make an accused yield certain information, the latter concerns the drawing of adverse inferences when an accused fails to testify or to answer questions (Ashworth 2008).

This privilege is enshrined in the International Covenant on Civil and Political Rights, which stipulates that “in the determination of any criminal charge against him, everyone shall be entitled (…) not to be compelled to testify against himself or to confess guilt” (Art. 14(3)(g)). A similar provision can be found in the American Convention on Human Rights and in the Rome Statute of the International Criminal Court.^6^ Although the European Convention on Human Rights does not explicitly refer to the privilege against self-incrimination, the European Court of Human Rights (ECtHR) has repeatedly asserted that this principle is implied in the general right to a fair trial, which is guaranteed by Article 6 of the Convention.^7^ In the US, the Fifth Amendment protects against “coercion [to] prove [a] charge against an accused out of his mouth”. Interpreting this clause, the US Supreme Court introduced in 1966 the distinction between being compelled to provide *real or physical evidence* (which is allowed) and being forced to give self-incriminating *testimony* (which is forbidden).^8^

The ECtHR draws a more subtle distinction when it differentiates between compelling “real evidence which has an existence independent of the will of the suspect” (ex. documents acquired pursuant to a warrant, breath, blood and urine samples and bodily tissues for the purpose of DNA testing) and evidence which is not truly “independent of the will of the suspect”.^9^ Answers to questions are the most obvious examples of this second category because they are inconceivable without the will of the subject. However, in the case of *Funke v. France*, the ECtHR has considered that also being compelled to produce certain documents (in the case, bank statements from accounts in foreign banks, and which might serve to incriminate the individual for tax evasion), would amount to an infringement of the privilege.

Therefore, the lecture of the privilege made by the ECtHR can been understood in the sense that the key issue is not so much whether the evidence is real or oral (i.e. physical as opposed to answers to questions), but whether the evidence requires the *active co-operation* of the individual or not (Redmayne 2007). In other words, “the privilege only covers assistance from the suspect which could not be substituted by employing direct force” (Trechsel 2005).

If we accept this understanding of the privilege, the question then becomes whether the mere record of thoughts and memories -without any coerced oral testimony or declaration- is evidence that can be legally compelled, or whether this practice necessarily requires the ‘will of the suspect’ and therefore constitutes a breach of the privilege against forced self-incrimination. Unfortunately, it is extremely difficult to give a clear-cut answer to this dilemma. In our opinion, the issue has to be the matter of public discussion in order to find an adequate balance between the private and public interests at stake. The dilemma is particularly arduous because, on the one hand, it could be argued that thoughts and memories are purely internal operations that *per se* cannot be forced, and consequently the non-incrimination clause would not be applicable to them. However, on the other hand, if mind-reading techniques are allowed in criminal proceedings, there is in the long term the risk to completely water down the privilege against self-incrimination, especially if the techniques become more reliable and efficient than they are at present. People might still be formally protected against self-incriminatory oral statements, but not against the very source of such testimonies: their own thoughts. As Nita Farahany (2012) puts it, self-incrimination may occur *silently* just as aloud.

### The right to mental integrity

Intrusions into people’s brains cannot only result in a violation of their mental privacy, but may also have a direct impact on their neural computation and result in direct harm to them. Ienca and Haselager (2016) have introduced the notion of *malicious brain-hacking* to refer to neurocriminal activities that influence directly neural computation in the users of neurodevices in a manner that resembles how computers are hacked in computer crime. Focusing on brain-computer interface (BCI), they identify four types of malicious brain-hacking based on the various levels of the BCI cycle where the attack can occur. Three of these types, i.e. when the attack occurs at the level of measurement, decoding and feedback, may involve direct manipulation of a person’s neural computation. Malicious agents may add noise or override the signal sent to the device with the purpose of diminishing or expunging the control of the user over the application, or even hijacking the victim’s voluntary control. For example, a criminal actor could override the signal sent by the users and hijack the BCI-controlled device (e.g. smartphone, electronic wheelchair) without the user’s permission.

In this kind of cases, the users’ mental privacy and the protection of their brain data are not the only rights at risk. Rather, the physical and mental integrity of the victim are at stake too. In fact, the forced intrusion into and alteration of a person’s neural processes pose an unprecedented threat to that person’s mental integrity.

The right to personal physical and mental integrity is protected by the EU’s Charter of fundamental rights (Article 3), stating that “everyone has the right to respect for his or her physical and mental integrity.” Understandably, the Charter emphasizes the importance of this right in the fields of medicine and biology, because of the direct impact that biomedical technologies may have on people’s physical and mental integrity. The provision focuses in particular on four requirements: free and informed consent, the non-commercialization of body elements, and the prohibition of eugenic practices and human reproductive cloning. No explicit reference is made to neurotechnology-related practices. This silence is understandable if we consider that the Charter was adopted in 2000, when the discussion on the ethical and legal implications of neuroscience was at a very early stage. Today however, potential applications of neurotechnology open the prospects of impacting personal integrity in a manner that is comparable to that of genetics and other biomedical practices. For this reason, the normative framework should keep up with neurotechnological advances and extend the protection of people’s integrity to this new area.

We propose to fill this normative gap by calling for a reconceptualization of the right to mental integrity. In fact, while the ECHR and the EU Charter of Fundamental Rights consider mental integrity as a right to mental-health, pendant of the right of physical integrity understood as physical health, a more complex dimension of mental integrity is elicited by neurotechnology. Mental integrity in this broader sense should not only guarantee the right of individuals with mental conditions to access mental health schemes and receive psychiatric treatment or support wherever needed. In addition to that, it should also guarantee the right of all individuals to protect their mental dimension from potential harm.

This reconceptualized right should provide a specific normative protection from potential neurotechnology-enabled interventions involving the unauthorized alteration of a person’s neural computation and potentially resulting in direct harm to the victim. For an action X, to qualify as a threat to mental integrity, it has to: (i) involve the direct access to and manipulation of neural signaling (ii) be unauthorized –i.e. must occur in absence of the informed consent of the signal generator, (iii) result in physical and/or psychological harm. As neurotechnology becomes part of the digital ecosystem and neural computation rapidly enters the infosphere, the mental integrity of individuals will be increasingly endangered if specific protective measures are not implemented.

Threats to mental integrity do not limit to malicious brain-hacking and similar illicit activities. Unauthorized alterations of a person’s neural computation could also emerge out of military applications of BCI technology for warfighter enhancement. Lebedev et al. have described that a neurologically controlled prosthetic could send tactile information back to the brain in nearly real time by using intracortical microstimulation (ICMS), essentially creating a “brain-machine-brain interface” (Lebedev et al. 2011). Such interventions may directly modify neurological activity and can be used to exert some degree of control over ground troop soldiers. For example, the Committee on Opportunities in Neuroscience for Future Army Applications of the National Research Council of the National Academies has investigated the use of portable technologies such as near infrared spectroscopy (NIRS) to detect deficiencies in a warfighter’s neurological processes and utilizing transcranial magnetic stimulation (TMS) to suppress or enhance individual brain processes (National Research Council 2009). Similarly, mental integrity rights should be included among the rights of war prisoners to prevent the use of invasive brain-washing interventions.

Brain stimulation is an additional domain where the right to mental integrity may play a role. With the growing number of portable neurostimulators available on the market or assembled do-it-yourself devices, the risk that people may misuse these devices with consequent negative impact on their neural functioning should be avoided. For example, while consumer-grade transcranial direct current stimulation (tDCS) are designed to safely function in a certain frequency band, little safeguards prevent users or third persons from manipulating the device’s frequency.

The medical domain is not exempt from the possible application of the right to mental integrity. Invasive neurotechnology interventions such as deep-brain stimulation (DBS) involve the alteration of the patient’s neural processing by electrode-delivered electrical impulses. While this procedure provides therapeutic benefits for otherwise treatment-resistant neurological patients, there is also the potential for neuropsychiatric adverse-effects including apathy, compulsive behavior and hallucinations (Mackenzie 2011). In addition, being a surgical procedure, there is a risk of infection, bleeding and rejection of the implanted neurostimulator. Therefore, although in such medical procedure informed consent is always obtained based on minimal medical ethics requirement, still there is a risk that the alteration of neural computation enabled by DBS may cause a disproportionate harm as compared to the therapeutic benefit. This high potential for adverse effects is the reason why, although having proved some effectiveness in the treatment of conditions such as obesity and anorexia nervosa, DBS is still not approved by the Food and Drug Administration (FDA) for the treatment of those conditions. In this context, mental integrity rights stand to prevent from harm, absolutely conceived, but to prevent to a disproportionate relative harm compared to the potential therapeutic benefit.

Finally, the growing field of memory engineering will likely represent a paramount challenge to the right to mental integrity. Several techniques have been developed to engineer (e.g. boost or selectively erase) memories from a person’s mind. For example, Nabavi and colleagues used an optogenetics technique to erase and subsequently restore selected memories by applying a stimulus via optical laser that selectively strengthens or weakens synaptic connections (Nabavi et al. 2014). While they have not reached yet the level of human experimentation, these findings may hold big potential for the treatment of such diseases as Alzheimer’s and post-traumatic stress disorder (PTSD). At the same time, however, the misuse of these techniques by malevolent actors may generate unprecedented opportunities for mental manipulation and brain-washing. For example, criminally motivated actors could selectively erase memories from their victims’ brains to prevent being identified by them later on or simply to cause them harm. On the long term-scenario, they could be used by surveillance and security agencies with the purpose of selectively erasing dangerous, inconvenient from people’s brain as portrayed in the movie *Men in Black* with the so-called *neuralyzer*. The potential motives of illicit memory alteration are various, including increasing national security or exerting control over individuals or groups.

Like the right to mental privacy, also the right to mental integrity may not be absolute. For example, it might be argued on utilitarian grounds that controlled and temporary violations of the right to mental integrity should be allowed as a form of moral enhancement for persistent violent offenders. For example, Persson and Savulescu (2008) have argued that if safe and effective biomedical moral enhancements were developed then they should be compulsory (Persson and Savulescu 2008). Similarly, Ellegaard and Kragh (2015) have argued that it is not only morally permissible, but morally required to force persistent violent offenders to undergo morally enhancing treatments provided the demonstrated effectiveness of such interventions. These possible exceptions to the right to mental integrity would obviously require broad societal discussion to determine whether –and when– such compulsory manipulations of the deepest dimension of the self could be justified for the greater benefit of society.

While taking a position in the long-standing debate over moral enhancement is beyond the scope of this paper, it is important to consider that the postulation of the rights to mental privacy and mental integrity does not *ipso fact*o implies the absolute character of these new rights.

### The right to psychological continuity

In addition to mental privacy and mental integrity, also people’s perception of their own identity may be put at risk by inadequate uses of emerging neurotechnology. As we have seen in the first section, neural devices can be used not exclusively for monitoring brain signals but also for stimulating or modulating brain function. For example, transcranial direct current stimulation (tDCS) devices apply constant, low current delivered to the brain area of interest via electrodes on the scalp with the purpose of modulating brain function. Since it causes neuron’s resting membrane potential to depolarize or hyperpolarize, this stimulation causes alterations in brain function that are potentially beneficial for patients. Transcranial magnetic stimulation (TMS) and deep brain stimulation (DBS) open the possibility of intervening into brain function even more substantially. Given the increasing therapeutic effectiveness of tDCS, TMS and DBS, and the rapid advancement of the technology, brain stimulation devices are likely to expand to wider psychiatric groups and, in the case of the first two ones, also to the general population.

However, changes in brain function caused by brain stimulation may also cause unintended alterations in mental states critical to personality, and can thereby affect an individual’s personal identity (Decker and Fleischer 2008). In particular, it has been observed that brain stimulation may have an impact on the psychological continuity of the person, i.e. the crucial requirement of personal identity consisting in experiencing oneself as persisting through time as the same person (Klaming and Haselager 2013). Several cases have been reported in the scientific literature in which DBS has led to behavioral changes such as increased impulsivity and aggressiveness (Frank, Samanta, Moustafa, and Sherman 2007; Sensi et al. 2004) or changes in sexual behavior (Houeto et al. 2002). A study involving patients treated with DBS showed that more than half of them articulated a feeling of strangeness and unfamiliarity with themselves after surgery (“I do not feel like myself anymore”; “I feel like a robot” or “I have not found myself again after the surgery”) (Schüpbach et al. 2006). More recent studies have evidenced personality changes in the direction of increased impulsivity (Lewis et al. 2015; Pham et al. 2015). In parallel, memory engineering technologies may impact a person’s identity by selectively removing, altering, adding or replacing individual memories that are relevant to their self-recognition as persons.

Surely it is an empirical question to determine the frequency and magnitude of these psycho-behavioral changes and it is a question for criminal and tort law to assess the impact of these changes on liability and responsibility. But the question we are interested in here is whether such personality changes induced by neurostimulation or memory manipulating technology could constitute in some circumstances a violation of a basic human right. This might theoretically be the case, for instance, if the patient is legally incompetent (for instance, a child) and the personality change turns out to be psychologically disturbing for him or her. In such circumstances, if the patient’s legal representatives refuse to consent to the removal of the device on the grounds that it has reduced the neurological disorder symptoms, they could be regarded as acting against the individual’s right to psychological continuity.

However, threats to this right are more likely to happen outside clinical settings. For instance, in the context of intelligence and military agencies, it has been reported that over the last decades violations of human rights might have taken place in experiments involving brain electrodes, LSD, hypnosis, the creation of Manchurian candidates,^10^ the implantation of false memories and induction of amnesia. Many of these experiments were conducted on unwitting civilians and in the absence of any external review, or representation for the experimental subjects, or any meaningful follow-up (Ross 2007). The new knowledge and technologies in the field of neuroscience clearly offer new and more efficient possibilities for carrying out unconsented personality changes. For example, Pycroft et al. (2016) recently reported the concern that brain implants like DBS are vulnerable to attack by third parties who want to exert malicious control over the users’ brain activity. They called this risk of modification of a person’s brain activity through unauthorized use of neurodevices by third parties ‘brainjacking’ (Pycroft et al. 2016). Negative consequences of brainjacking include (i) information theft, which would result in a violation of the right to mental privacy; (ii) cessation of stimulation, draining implant batteries, inducing tissue damage, and impairment of motor function, which would result in violations of the right to mental integrity. However, some possible consequences of brainjacking such as alteration of impulse control, modification of emotions or affect, induction of pain, and modulation of the reward system could be achieved even in absence of any violation of mental privacy and integrity. In those circumstances of unauthorized modification of the cognitive-emotional-affective dimension a different type of human right violation seems to be at stake: the violation of the right to psychological continuity.

In short, the right to psychological continuity ultimately tends to preserve personal identity and the coherence of the individual’s behavior from unconsented modification by third parties. It protects the continuity across a person’s habitual thoughts, preferences, and choices by protecting the underlying neural functioning. As Paul Tiedemann points out, we understand ourselves as personal unities and as subjects and source of attitudes as long as these attitudes have a minimum level of coherence. This is why a serious lack of coherence makes it impossible to understand oneself (Tiedemann 2016).

The right to psychological continuity can be seen as a special neuro-focused instance of the right to identity. The right to identity was developed by the European Court of Human Rights (ECtHR) from the right to private life included in Article 8 of the European Convention on Human Rights.^11^ As we have seen in the first section, Article 8 protects against unwanted intrusion and provides for the respect of an individual’s private space. However, privacy and personal identity should be distinguished. What the right to psychological continuity aims to prevent is not the unrestricted access to brain information but the induced alteration of neural functioning.

The UDHR also addresses the right to have and develop a personality. Article 22 states: “Everyone is entitled to the realization of the rights needed for one’s dignity and the free development of their personality.” In addition, Article 29 states: “[e]veryone has duties to the community in which alone the free and full development of his personality is possible.” According to Mănuc (2012), personality rights can be defined as those expressing the quintessence of the human person, and are intrinsic to being human. In here analysis, these rights recognize the “spirit” within an individual and have developed from the issues of privacy. It is questionable, however, if current personality rights are well-equipped to address the problem of stimulation-induced alterations in one’s personality.

However, it is questionable whether current personality rights can fully account for the threats posed to psychological continuity. In fact, while this family of rights protects the translation of mental states into action, psychological continuity guarantees protection at an antecedent level: at the level of raw neural functioning. In the risk scenario presented above, misused brain stimulation does not impact the link between mental processes and action, i.e. the expression of mental states, but the mental processes themselves. To provide this more intimate level of protection, there is a need for a new right that preserves the continuity of a person’s mental life from external abusive alteration or disruption.

The right to psychological continuity is closely related to the right to mental integrity, and may factually overlap with it. Both rights stand to protect people from abusive and unconsented alterations of their mental dimension. However, they differ to the extent that the right to psychological continuity also applies to emerging scenarios that do not directly involve neural or mental harm. In contrast, as we have seen in the previous section, the presence of harm is a necessary condition for an action to qualify as an offence to a person’s mental integrity.

To appreciate this difference, it is important to consider that psychological continuity could be threatened not only by misused brain stimulation but also by less invasive, even unperceivable interventions. A good example is unconscious neural advertising via neuromarketing. As we have seen in the first section, neuromarketing companies are testing subliminal techniques such as embedding subliminal stimuli with the purpose of eliciting responses (e.g. preferring item A instead of B) that people cannot consciously register. This has raised criticism among consumer advocate organizations, such as the Center for Digital Democracy, which have warned against neuromarketing’s potentially invasive technology. Jeff Chester, the executive director of the organization, has claimed that “though there has not historically been regulation on adult advertising due to adults having defense mechanisms to discern what is true and untrue”, it should now be regulated “if the advertising is now purposely designed to bypass those rational defenses” (Singer 2010). We argue that a right to psychological continuity can provide the conceptual basis be a viable solution to overcome the problems addressed by Chester.

Potential threats that could be prevented by the right to psychological continuity also include new forms of brain-washing. Holbrook et al. (2016) used transcranial magnetic stimulation (TMS) to neuromodulate the brain regions responsible for social prejudice and political and religious beliefs. Their results show that by temporarily turning off the posterior medial frontal cortex via TMS it was possible to make participants more positive towards criticisms to their country, than the participants whose brains were unaffected. Using the same technique, they could enhance the participants’ belief in afterlife. While their experiment was designed to mapping the precise neural mechanisms of high-level attitudes and beliefs, their results show that the same technique could be used to trigger a wide spectrum of alterations of a person’s attitudes and beliefs. Malicious agents, for example, could use neuromodulation to exert malevolent forms of mind control. These potentially include religious leaders and coordinators of religiously inspired terrorist groups who want to achieve effective indoctrination and recruitment of youngsters, as well as leaders of authoritarian regimes who want to enforce political compliance and prevent rebellion. More mildly, marketing companies could use these techniques to modulate customers’ preferences and attitudes towards their products.

Just like the previous two rights, it is a matter of discussion whether the right to psychological continuity should be considered absolute or relative. It could be argued that some neurotechnologically-induced personality changes could be tolerated with regard to persistent violent offenders (for instance, serial rapists, killers and pedophiles). The need to protect the public from potentially dangerous individuals who are very likely to reoffend if released would justify such measures. This would even be a good alternative for those individuals themselves, who could avoid in this way spending their whole lives in prison. However, extreme caution and broad public discussion is imperative before authorizing such intentional intrusions into people’s personality.

## Conclusions

The volume and variety of neurotechnology applications is rapidly increasing inside and outside the clinical and research setting. The ubiquitous distribution of cheaper, scalable and easy-to-use neuroapplications has the potential of opening unprecedented opportunities at the brain-machine interface and making neurotechnology intricately embedded in our everyday life. While this technological trend may generate immense advantage for society at large in terms of clinical benefit, prevention, self-quantification, bias-reduction, personalized technology use, marketing analysis, military dominance, national security and even judicial accuracy, yet its implications for ethics and the law remain largely unexplored. We argue that in the light of the disruptive change that neurotechnology is determining in the digital ecosystem, the normative terrain should be urgently prepared to prevent misuse or unintended negative consequences. In addition, given the fundamental character of the neurocognitive dimension, we argue that such normative response should not exclusively focus on tort law but also on foundational issues at the level of human right law.

In this context, we have suggested that emerging trends in neurotechnology are eliciting coordinate amendments to the current human right framework which will require either a reconceptualization of existing human rights or even the creation of new neuro-specific rights. In particular, we have argued that emerging collateral risks associated with the widespread use of pervasive neurotechnology such as malicious brain-hacking as well as hazardous uses of medical neurotechnology may require a reconceptualization of the right to mental integrity. In fact, although mental integrity is protected by the EU Charter of Fundamental Rights (Article 3), this right is conceptualized as a right to accessing and protecting mental health and is complementary to the right to physical integrity. We suggest that in response to emerging neurotechnology possibilities, the right to mental integrity should not exclusively guarantee protection from mental illness or traumatic injury but also from unauthorized intrusions into a person’s mental wellbeing performed through the use of neurotechnology, especially if such intrusions result in physical or mental harm to the neurotechnology user.

In addition to such reconceptualization, we have argued that the creation of neuro-specific rights may be required as a coping strategy against possible misuses of neurotechnology as well as a form of protection of fundamental liberties associated with individual decision-making in the context of neurotechnology use. With this respect, we have endorsed the recognition of a negative right to cognitive liberty as a right for the protection of individuals from the coercive and unconsented use of such technologies. In addition, as a complementary solution, we have proposed the recognition of two additional neuro-specific rights: the right to mental privacy and the right to psychological continuity. The right to mental privacy is a neuro-specific privacy right which protects private or sensitive information in a person’s mind from unauthorized collection, storage, use, or even deletion — in digital form or otherwise. In contrast to existing privacy rights, the right to mental privacy stands to protect information prior to any extra-cranial externalization (e.g. in verbal or printed format) as well as the generator of such information (a person’s neural processing). As such, it protects a person’s mental dimension as the ultimate domain of information privacy in the digital ecosystem. In coordination with that, the right to psychological continuity will protect the mental substrates of personal identity from unconscious and unconsented alteration by third parties through the use of invasive or non-invasive neurotechnology.

All these proposed neuro-focused rights are mutually linked and stand in an intimate family relationship. Being the substrate of all other freedoms, cognitive liberty in its positive sense is a prerequisite of all other neuro-focused rights. As such, it is to mental privacy, mental integrity and psychological continuity in a very similar relation as freedom of thought is to privacy, integrity and identity rights. However, in its negative sense of protection from coercive use, cognitive liberty can only partly account for unintended uses of emerging neurotechnology. In fact, illicit intrusions into a person’s mental privacy may not necessarily involve coercion, as they could be performed under the threshold of a persons’ conscious experience. The same goes for actions involving harm to a person’s mental life or unauthorized modifications of a person’s psychological continuity, which are also facilitated by the ability of emerging neurotechnologies to intervene into a person’s neural processing in absence of the person’s awareness.

This proposal of neuro-specific human rights in response to emerging advancements in neurotechnology is consistent with and a logical continuation of the proposal of developing genetic-specific human rights in response to advancements in genetics and genomics as set out by the *Universal Declaration on the Human Genome and Human Rights* (UDHGHR) and the *International Declaration on Human Genetic Data* (IDHGD).

Extensive future debate is required to test the normative solidity of this proposed expansion of the human right framework to the neurotechnology dimension. In parallel, future research is required to investigate the implications of such proposed human rights on other levels of law such as international humanitarian law, criminal law, tort law, property law and consumer law. Ideally, this debate should benefit from the active and cross-disciplinary participation of legal experts, neuroscientists, technology developers, neuroethicists and regulation bodies.

## Footnotes

1 For example, the website The Brain Stimulator offers a wide assortment of affordable tDCS devices, with prices ranging between 60$ and 200$. See: https://thebrainstimulator.net/shop/

2 Speech at Panel on “What If: Your Brain Confesses?” World Economic Forum - Annual Meeting, Davos, 20–23 January 2016. Available at: https://www.weforum.org/events/world-economic-forum-annual-meeting-2016/sessions/what-if-your-brain-confesses

3 See http://ec.europa.eu/justice/data-protection/reform/index_en.htm

4 The expression “reasonable expectation of privacy” was coined by the US Supreme Court in 1967 to distinguish legitimate police searches and seizures from unreasonable ones in the light of the Fourth Amendment that protects privacy rights.

5 “In the exercise of his rights and freedoms, everyone shall be subject only to such limitations as are determined by law solely for the purpose of securing due recognition and respect for the rights and freedoms of others and of meeting the just requirements of morality, public order and the general welfare in a democratic society” (UDHR, Article 29.2).

6 American Convention on Human Rights, art. 8(2)(g): “Every person accused of a criminal offense has the right (…) not to be compelled to be a witness against himself or to plead guilty”; Rome Statute of the International Criminal Court, art. 55(1)(a): “In respect of an investigation under this Statute, a person: (a) Shall not be compelled to incriminate himself or herself or to confess guilt”. Paragraph 2(b) of the same Article 55 adds that the person suspected of having committed a crime has the right “to remain silent, without such silence being a consideration in the determination of guilt or innocence”.

7
*Funke v. France,* ECtHR 1993, A n° 256-A; *John Murray v. United Kingdom,* ECtHR 1996-I.

8 US Supreme Court, *Miranda v. Arizona*, 384 U.S. 436 (1966).

9
*Saunders v. United Kingdom,* ECtHR 1996-VI, para 69.

10 The expression “Manchurian candidate” refers to “a person who is (or is believed to be) brainwashed into becoming a subversive agent, especially an assassin” (Oxford Dictionary). The expression was popularized by the 1962 film *The Manchurian Candidate*, adapted from the 1959 novel of the same name by Richard Condon.

11
*Goodwin v United Kingdom* ECtHR (2002) 35, 18 at 90.
